# State Board of Veterinary Examiners

**Published:** 1902-06

**Authors:** 


### STATE BOARD OF VETERINARY EXAMINERS.

Ohio State veterinary law has been sustained by a decision of the lower court, and four illegal practitioners are now under indictment.

At the organization meeting of the New Jersey State Board of Veterinary Medical Examiners, held at the State House in the city of Trenton, on May 5th, Dr. William Herbert Lowe, of Paterson, was elected President; Dr. Whitfield Gray, of Newton, Secretary; and Dr. T. E. Smith, of Jersey City, Treasurer.

